# Self and Charity Advertising

**Published:** 1910-02-12

**Authors:** 


### The Hospital

A Journal of

The Medical Sciences and Hospital Administration.

New Series. No. 156, Vol. VI. [No. 1228, Vol. XLVII.] SATURDAY, FEBRUARY 12, 1910.

### Self and Charity Advertising.

The genuine interest which the King and many members of his family have continuously displayed in charitable effort, especially for the hospitals, has done an immense service to the cause of the sick. To it must also be attributed, in a measure, the feverish, hungry rush which a number of people have made in recent years to advertise themselves as kings of this or that, by riding into notoriety on the back of one or more established charities or organisations. Many of these self-advertisers are properly regarded as a curse by the real workers, who are the backbone of charity, and whose labours are quietly and continuously carried on, unknown for the most part to the public. In these days of personal journalism the self-advertiser may readily arouse admiration in the minds of the unthinking majority of casual readers, who worship publicity of all kinds. They indeed hold that the man who advertises himself sufficiently must ultimately excite an interest in the charity he selects to ride upon into notoriety. If this were true in fact, the fate of the self-advertiser would be identical with that of the lady who went for a ride on a tiger. Unfortunately, experience proves that the exact contrary is the case, for it is the institution, not the individual, that ultimately suffers.

The difference to an institution where its moving spirit or spirits are either (1) self-advertisers or (2) silent workers is that the former put themselves forward first, not the charity, and that the latter put the charity first in everything and efface themselves. If the self-advertiser has ability the institution may benefit for a period under his control, but if his abilities are small it never really benefits at all. The results, however, to the institution are the same in both cases—*i.e.* ultimate set-back. The abnormal exaltation of the self-advertiser in recent years has proved rather costly to certain institutions. We know hospitals which are suffering from this cause, where there has been a serious diminution in revenue, or where the building up of permanent revenue has been imperilled, if it has not even been stayed. Hence one or more of the great Funds might helpfully test the causes of the marked shortage in revenue in a few notable cases. Charity advertising, as it called, is undoubtedly an art, known only to the few. It has happened within our knowledge that, where the chief officials have mastered the art, their efforts have been paralysed by the advent of an ambitious individual in an honorary capacity who thirsted for notoriety or fame, or whatever name properly describes the motive, and so brought himself and his authority always forward to the exclusion of many other necessary and better things. On the ultimate withdrawal of these influences, the financial position of the charity had to be taken in hand *de novo*, and much lost ground had to be recovered. These things should not be. Hospital committees especially should be masters in their own house, and no individual among them should be permitted to usurp authority, keep his name always before the public, crush out individual effort, and prevent the enforcement of a wise, just, and liberal policy in relation to the staff and the business of the charity.

Every voluntary charity should have a trained staff, whose duty it should be to maintain and increase the revenue, and to keep it adequate to meet the needs and claims upon it. The work of this staff should be controlled by a small, active finance committee of business men, who should intelligently supervise the advertising in all its branches and so prevent much of the useless expenditure of money upon advertisements in a class of publication which still flourishes in a modernised form, though it was exposed in The Hospital of November 20, 1886, pages 122-3. It is scarcely credible, but it is a fact, that there are publications of this class to-day which will give a portrait of the chief official or chairman, with an article on the charity, when advertising space is taken to the value of a certain sum. It is part of our routine to go through the columns of these publications, and it amazes us to see the amount of money which is still uselessly spent on this type of advertisement. It was referred to at a recent meeting of the Sphinx Club, the members of which, and presumably the guests also, are interested in all branches of advertising.

We cannot agree with some of the views put forward at that meeting. We do not believe in the class of advertisement which records in the largest type that five billion drops of milk or trillions of breadcrumbs are annually consumed by the patients of a particular hospital. This sort of rubbish properly disgusts those who have it in their power to give most liberally to hospitals, and tends to belittle rather than to raise the popularity of a great hospital, the affairs of which should be conducted with dignity and quiet strength. At one great London hospital the name of the Treasurer is seldom seen in the papers, but this Treasurer has done more in the way of raising money from the public for his hospital than probably any worker for a single charity since the world began. He is a public man, too, of high repute, but few of the public, and a remarkably small number of hospital people, realise the magnificence of his work and its actual results in pounds sterling. This is the proper spirit, and what that spirit can do for the hospitals is eloquently testified by the success of the King's Hospital Fund and the League of Mercy. Charity advertising to be successful should result in the association together of an ever-increasing number of quiet workers, who take a growing interest in the development of the particular charity with which they identify themselves. Thus they may continually increase its resources, promote the efficiency of its work, and extend the area of its influence for good. What our hospitals need at present is more zealous workers to the exclusion of less desirable elements.

